# Liquid-Crystal-Based Electrically Tuned Electromagnetically Induced Transparency Metasurface Switch

**DOI:** 10.1038/s41598-017-17612-7

**Published:** 2017-12-12

**Authors:** Hang Su, Hao Wang, Hua Zhao, Tingyu Xue, Jingwen Zhang

**Affiliations:** 1 Department of Physics, Harbin Institute of Technology, Harbin, 150001 China; 2 Key Laboratory of Micro-Optics and Photonics Technology of Heilongjiang Province, Harbin, 150001 China

## Abstract

In this study, a structure to realize a switchover between two different responses of electromagnetically induced transparency (EIT) was designed and implemented by simulation. Taking advantage of the anisotropy in the structure and the coupling between the radiative and dark elements, a metasurface switch with modulation depth of over 85% between orthogonal polarization incident light illuminations was demonstrated. The key mode switchover between the “on” and “off” states was achieved by electrically changing the dressing light polarization with a liquid crystals layer pre-aligned with a mature technology, without changing the incident light and an expected and reversible transition from an EIT-like spectrum to a strong spectral dip was observed. The modulation in the EIT switch fabricated with the proposed straightforward approach is a promising tool to control the groping velocity delay.

## Introduction

A metasurface is a two-dimensional thin-film-based planar structure composed of resonant meta-atoms^1^, which holds potential for fabricating flat, ultrathin components with a variety of optical functionalities, such as polarization manipulation, sensing, dispersion engineering and emission control^2^. Among them, the ability to create arbitrary wavefronts by spatially distributing phase discontinuities has brought breakthroughs in wavefront engineering^3,4^, which has led to rapid advancements in the field of phase manipulation such as beam deflection and holography by using gradient metasurfaces^5–8^. Meanwhile, periodically structured metallic metasurfaces offer opportunities for tailoring transmission, reflection and absorption in different spectral regimes by changing materials and structures^9^. These metasurfaces can be fabricated with mature nanotechnologies such as electrical beam lithography (EBL) and focused ion beam lithography (FIB), and exhibit a broad spectrum of applications to replace many traditional bulk optical devices^10,11^. In these multifarious metasurfaces, electromagnetically induced transparency (EIT)^12,13^ based on local surface plasmons has generated growing interest owing to the narrow transparent peak within a broader reflection spectrum which results from the coupling effect between the radiative and dark elements in the EIT metasurface. Many applications such as highly sensitive sensing^14^, quantum information^15^, slow light^16,17^, and plasmonic resonance switch^18,19^ have been developed based on EIT over the past years. One of the most significant parameters in EIT is the coupling efficiency, which affects the resonance modes of EIT-like metasurfaces, namely transparency and dip/peak doublet positions and group velocity. Thus, the transmission and reflection spectra can be tailored by tuning coupling efficiency to spatial structure dimensions.

Unfortunately, the “static” metasurfaces are not applicable in multifunctional devices. Recent works have shown that metasurfaces or metasurface systems are combined with other materials to become dynamical and to realize artificial controlling^1^; several methods were initiated by utilizing semiconductors^20^, phase changing materials^21^, graphene^22^ and liquid crystals^23–27^ (LCs). Considering these diverse approaches, the choice of a LC layer (or LC mixture) is very attractive because of its unique optical properties, in particular nonlinearity^27–29^, large optical anisotropy and birefringence^30^, which can be externally controlled by temperature^31^, incident light, and electric or magnetic fields^32,33^. A LC layer (especially nematic LCs) can be integrated into metasurfaces and plasmonic systems to bring additional active functionalities^24,34^, especially spectral response by controlling stiction in nano-electro-mechanical system^35^. With the spectral shift induced by the change of LC orientation, absorption modulation^32^ can be realized and hence, different colors can be displayed by a functional metasurface^36^. In addition, it can also create a switch effect in the spectrum^9,34,37^.

In this paper, we introduce a design of electrically controlled EIT metasurface loaded with nematic LC, which allows us to change the spectral resonance response of the metasurface in the NIR region by tuning an external electric field, under an unchanged incident light. The LC molecules have a strong anchoring force on the neighboring surface when no external field is applied, which is defined as the “off” state in our work. Correspondingly, if an external electric field is applied across the LC layer, the directions of the LC molecules reorient along the field direction and thus exhibit different optical properties from the original state, which is defined as the “on” state. In other words, the orientation of the LC molecules and hence the polarization of the incident beam out of the LC layer can be controlled by an external electric field. In the proposed design, the incident light traverses successively through the upper electrode, pre-alignment layer, LC layer, metasurface, and bottom electrode. The incident light is modulated after passing across the LC layer and rotates by 90° with polarization direction to the metasurface in the “off” state, or remains unchanged in the “on” state. Subsequently, the modulated light has a strong interaction with different elements of the metasurface and hence gives rise to different responses and absorption-line-types of EIT phenomenon. Meanwhile, the entirely different response modes support two kinds of linear susceptibility, which include two imaginary-part line types. As a consequence, the group velocity could be totally different under two resonance modes.

## Results

### Characterization of the LC-metasurface cell

**Figure 1:**
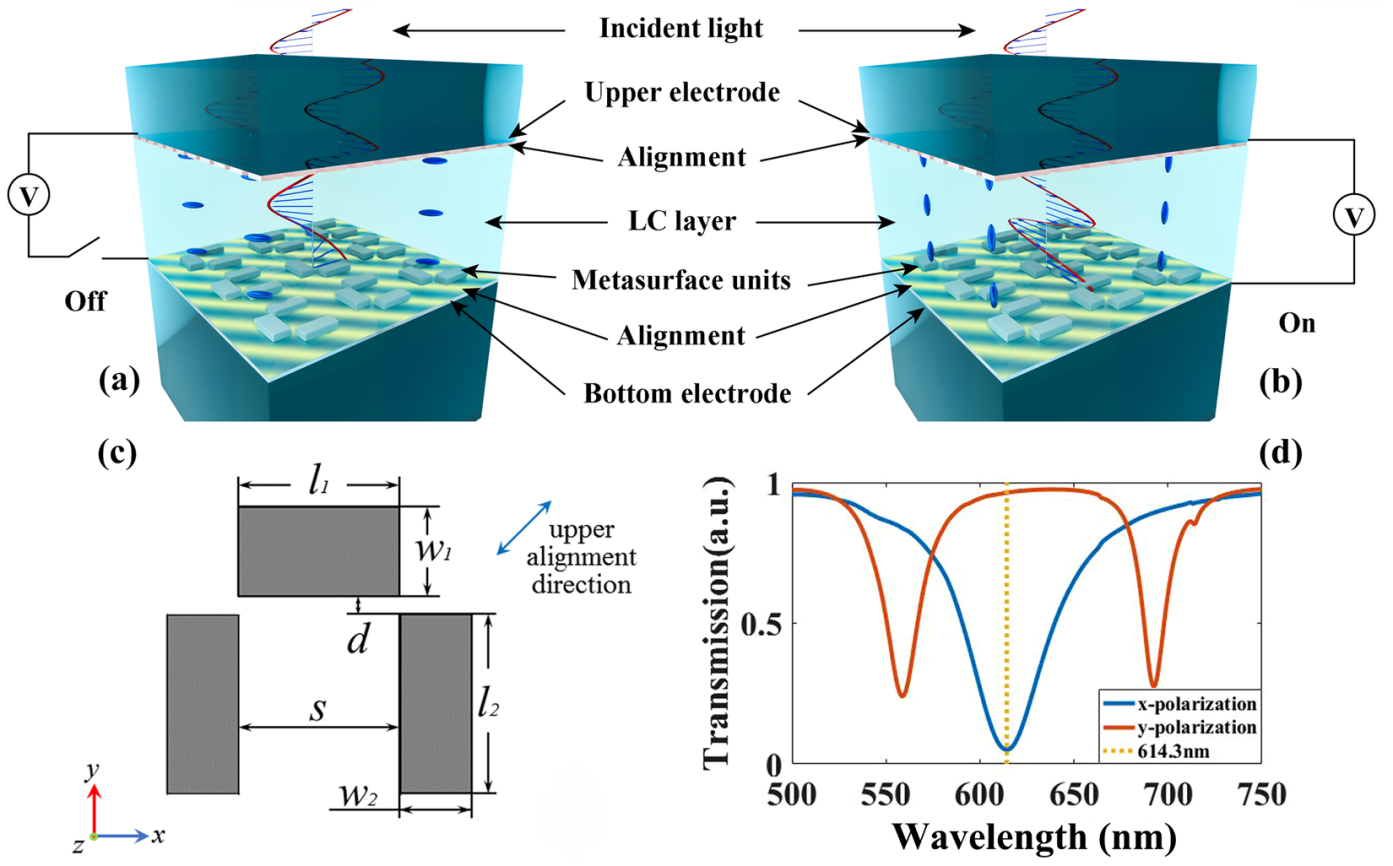
Schematic diagram for the EIT metasurface and LC sections at (**a**) the “off” state and (**b**) the “on” state. (**c**) Planar structural representation of a metasurface unit. (**d**) Numerical result of the metasurface responses under the x- and y-polarized incident light without the LC layer.

The LC-metasurface cell shown in Fig. 1(a,b) can be divided into two functional sections: the EIT metasurface section and the LC modulation section. The EIT metasurface units are designed with a lattice of 400 nm on a glass substrate covered with an indium-tin-oxide (ITO) layer of 20 nm in thickness as the bottom electrode. The dimensions of both the “dark plasmonic atom” and “radiative atom” are adjusted to respond in the appropriate spectral regime. For each metasurface unit, the “radiative atom” is a silver strip with lateral dimensions *l*_1_ = 90 nm, *w*_1_ = 50 nm. The “dark plasmonic atom” consists of a pair of silver strips whose lateral dimensions are *w*_2_ = 40 nm and *l*_2_ = 100 nm, 90 nm apart [Fig. 1(c)]. The separation of radiative element and dark elements is 10 nm, to adjust coupling of the single EIT metasurface unit. Both the radiative element and dark elements have the same thickness of 20 nm.

The transparent, conductive ITO layer under the silver metasurface units serves as the bottom electrode and the upper ITO glass substrate serves as the top electrode of the LC layer. In the simulation, we neglect the absorption of top electrode and alignment layers owing to their high transparency in the spectral regime of interest, small thickness, and lack of modulation to polarization. For the bottom electrode, we set a layer of ITO and an absorption layer in simulation model with the thickness of 20 nm and 5 nm correspondingly, which will be explained in the following part. The incident light polarization is fixed along x-axis. In the “on” state, the molecules in the LC layer are aligned along the *z*-axis and uniform in the *x-y* plane; thus, we can regard the LC layer as a uniaxial crystal with the optical axis parallel to the *z*-axis. The incident light travels along the optical axis and no polarization modulation is introduced. Therefore, an alignment layer is necessary for the LC-metasurface cell to obtain the transition from the x-polarization to y-polarization^30^ in the “off” state. This transition will be explained in detail. Two approaches for pre-alignment are used in our system. The upper pre-alignment layer is a 200 nm-thick layer of polyvinyl alcohol (PVA) in the *x*-*y* plane on top of the ITO layer, which is preprocessed by brushing mechanically on the surface. It should be noted that the brushing direction is 45° to the *x*-axis [shown in Fig. 1(c)]; thus, the pre-alignment of LC molecules is entirely in the *x*-*y* plane at an angle of 45° both to the *x*- and *y*-axes. The bottom metasurface is spin-coated with sulfonic azo dye (SD1) as the bottom alignment layer, considering the frangibility of the metasurface units. The SD1 layer has a thickness of 5 nm and is polarization photosensitive^38^. Hence, by exposing it to a polarized UV light source, the SD1 molecules tend to reorient perpendicular to the polarization orientation. In our system, the UV light source polarization orientation is 135° to the *x*-axis, and thus, the SD1 molecules orientation is 45° to the *x*-axis, which allows the control of the LC alignment in the same manner as that of the bottom pre-alignment.

We choose nematic LC (4,4′-n-pentylcyanobiphenyl, 5CB) in the LC-metasurface cell and treat the aligned LC molecules as a block of uniaxial crystal with different optical axes after pre-alignment and applying an external electric field (for example, the refractive index matrix can be treated as [1.5,1.5,1.7] when the optical axis is along the *z*-axis at room temperature^39^). The LC layer supports a refractive index difference, thus common LC material (E7) is also another selection. Under this condition, the phase delay between the ordinary and extraordinary portions of the incident light could be realized after passing through the LC layer. The thickness of LC layer is 2.3 μm, which is calculated to obtain optimal polarization modulation at 932.5 nm. The length of LC layer should be adjusted according to the refractive index difference in this spectra range.

In our design, the transmission change between the two states can serve as an excellent electrically controlled optical switch. Among all different functional layers, the LC layer provides a mode change to the metasurface under the same incident light provided by the external electric field or pre-alignment layer. In other words, the polarization modulated light gives rise to different type of resonances at the surface of the metasurface units, which results in a switching function at 932.5 nm.

### Switching of metasurface resonances

The transmission switching is based on two entirely different resonance modes of the EIT-like metasurface under x- or y-polarized incident lights due to the anisotropy of the geometry of metasurface units. The connection between EIT atomic and metamaterial systems was first shown in N. Papasimakis’ work^40^, which demonstrates a classical analog of electromagnetically induced transparency in a planar metamaterial. Here, we determine the transmission calculated from the EIT atomic theory under two different parameter situations, which is an analogy to our simulation. The details of EIT atomic theory analysis are presented in the supplementary information.

1 $${\chi }^{(1)}(-{\omega }_{p},{\omega }_{p})=\frac{{|{\mu }_{13}|}^{2}\xi }{{\varepsilon }_{0}\hslash }\times [\frac{4\delta ({|{{\rm{\Omega }}}_{c}|}^{2}-4\delta {\rm{\Delta }})-4{\rm{\Delta }}{\gamma }_{21}^{2}}{{|{|{{\rm{\Omega }}}_{c}|}^{2}+({\gamma }_{31}+i2{\rm{\Delta }})({\gamma }_{21}+i2\delta )|}^{2}}+i\frac{8{\delta }^{2}{\gamma }_{31}+2{\gamma }_{21}({|{{\rm{\Omega }}}_{c}|}^{2}+{\gamma }_{21}{\gamma }_{31})}{{|{|{{\rm{\Omega }}}_{c}|}^{2}+({\gamma }_{31}+i2{\rm{\Delta }})({\gamma }_{21}+i2\delta )|}^{2}}]$$

**Figure 2:**
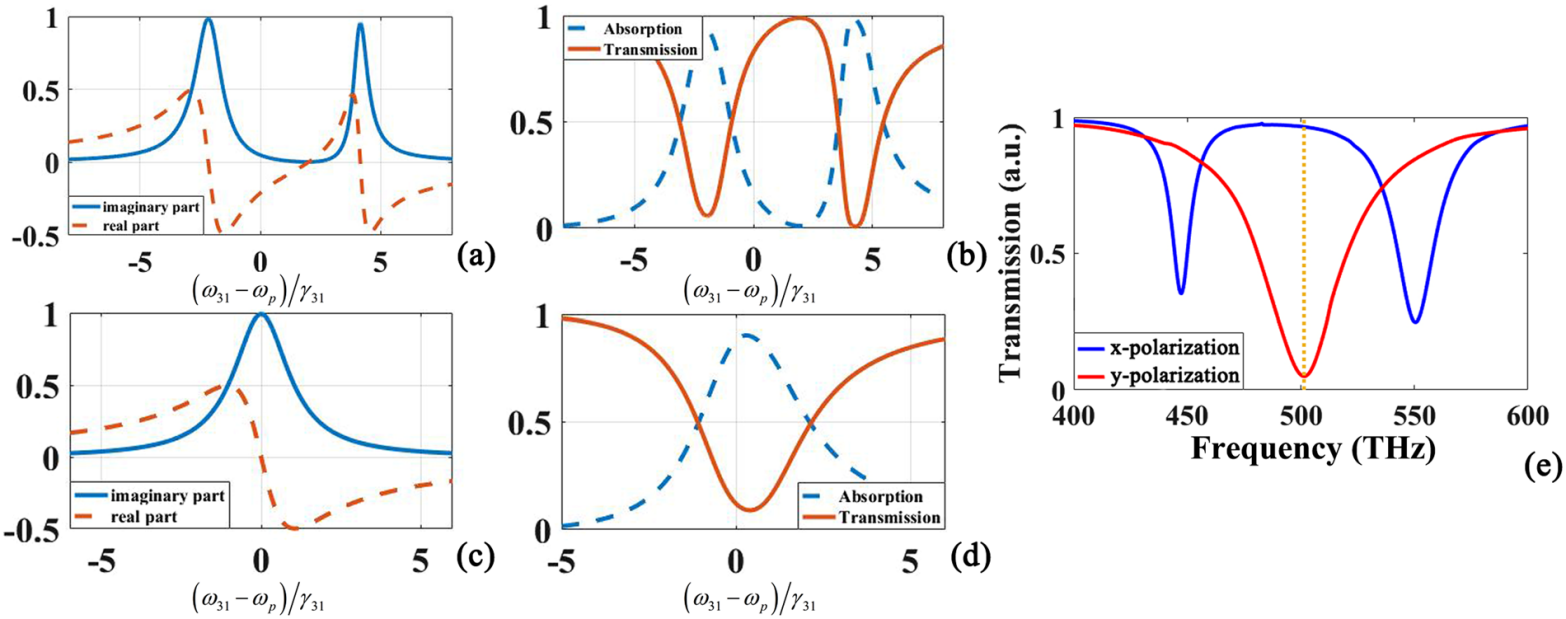
Transmission contrast between EIT atomic theory [(**a**–**d**)] and simulation results (**e**). The red line in (**b**) and (**d**) shows the transmission calculated from linear susceptibility and they are analogies of different polarization incident light responses (blue line and red line) in (**e**) respectively.

**Figure 3:**
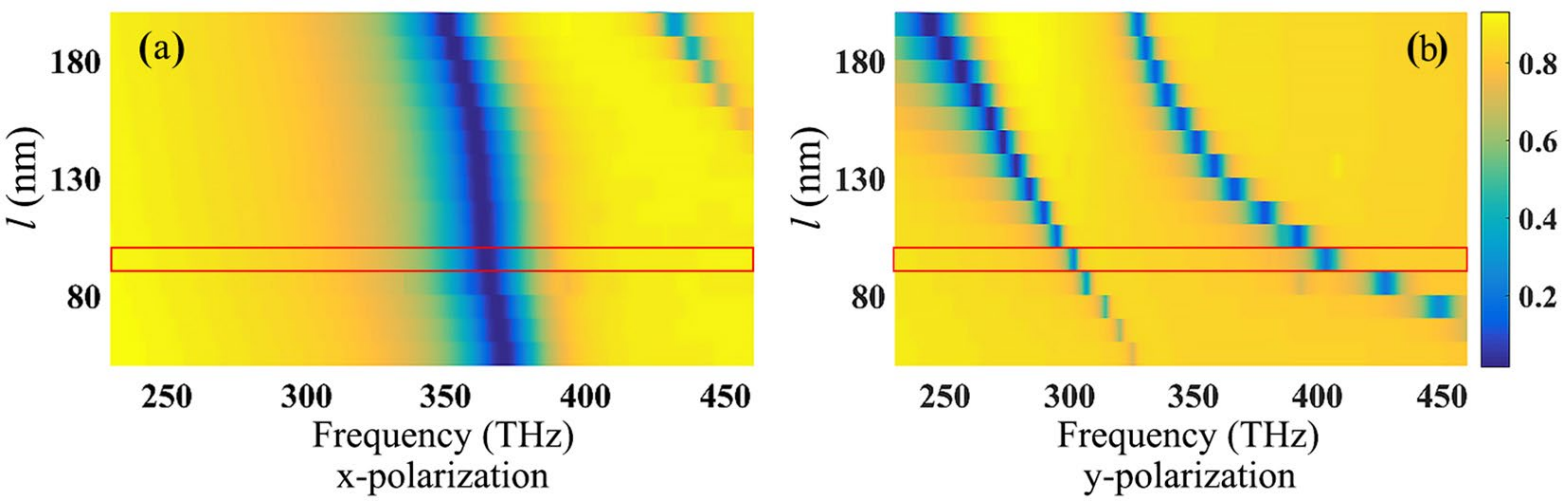
Transmission spectrum with different length of *l*_1_ in Fig. 1(c). The responses shows an obvious relationship with polarization. The dimensions in red line was selected in our simulation work due to the proper positions of resonance peaks.

Figure 2 show the contrast between theory and simulation for the case of transmission. The line type of linear susceptibility’s real parts and imaginary parts calculated from the supplementary information are totally different by adjusting parameters in Eq. 1 (Eq.S5 in Supplementary Information), which are different responses’ analogy of EIT-like metasurface with two orthogonality polarized light. We can easily calculate the absorption and refractive index from linear susceptibility’s real parts and imaginary parts and there is an obvious analogy between the simulation results and theory calculation. Meanwhile, there are many parameters in the EIT-like metasurface controlling the transmission spectra and the length of radiative element *l*_1_ in Fig. 1(c) is swept from 80 nm to 200 nm shown in Fig. 3(a,b). Thus, we can control the transmission spectrum position tailor the line as desired with changing the structure dimensions. The transmission sweep chart shows a strong dependency with incident light polarization and the parameters we selected in simulation is marked with red line.

The principle of switching between different resonances via a twisted LC-cell was first described and realized by the Southampton group^41^ and we use this concept for reference in our simulation. The application or lack thereof of an external electric field is defined as the “on” state or “off” state, respectively, and these conditions are shown in Fig. 1(a,b). The two electrodes are placed parallel and their normals are along the *z*-axis; thus, the external electric field direction is along the *z*-axis in the “on” state. When the external electric field is applied on the LC layer, the directors of most LC molecules reorient along the field direction (*z*-axis). However, a very thin layer of LC molecules adjoining the brushed layer will remain in the same orientation even in the “off” state, due to the strong surface anchoring^33^. In our calculation, we neglect the anchored layer in the “on” state because the layer can be extremely thin when the external electric field is sufficiently strong, and thus, the influence of anchored layer the incident light is insignificant. In this situation, the LC layer can still be seen as a uniaxial crystal with optical axis along the *z*-axis and the permittivity tensor is expressed as the following,

2 $${\varepsilon }_{{\rm{on}}}\approx [\begin{array}{ccc}2.25 & 0 & 0\\ 0 & 2.25 & 0\\ 0 & 0 & 2.89\end{array}]$$

where we neglect dispersion because of little refractive index change for both n_o_ and n_e_^32,39^. The electric field of the incident light is perpendicular to the optical axis for the entire duration of propagation, which only results in a change in magnitude but no polarization variation.

In the “off” state, there is no voltage applied on the electrodes, which means that the orientation of each LC molecule depends only on the van der Waals forces experienced by the surrounding molecules and their morphology. The closest layer of molecules to the pre-alignment layer is anchored to the brushed direction, and affects the neighboring molecules that do not adjoin the brushed layer directly. As the van der Waals force in the LC layer is strong enough to hold all molecules in the brushed direction, which is aligned 45° to the *x*-axis, we regard the whole LC layer as a uniaxial crystal with the optical axis in the brushed direction in the *x*-*y* plane. The crystal permittivity tensor can be described as:

3 $$\begin{array}{rcl}{\varepsilon }_{{\rm{off}}}=\varepsilon ^{\prime} =(A)(\varepsilon ){(A)}^{-1} & = & [\begin{array}{ccc}\cos \,\theta  & \sin \,\theta  & 0\\ -\,\sin \,\theta  & \cos \,\theta  & 0\\ 0 & 0 & 1\end{array}]\,[\begin{array}{ccc}2.89 & 0 & 0\\ 0 & 2.25 & 0\\ 0 & 0 & 2.25\end{array}]\,[\begin{array}{ccc}\cos \,\theta  & -\,\sin \,\theta  & 0\\ \sin \,\theta  & \cos \,\theta  & 0\\ 0 & 0 & 1\end{array}]\\  & = & [\begin{array}{ccc}2.57 & 0.32 & 0\\ 0.32 & 2.57 & 0\\ 0 & 0 & 2.25\end{array}],\end{array}$$

where *ε* is the initial dielectric tensor for the LC layer at principal axes system and *A* is a rotation operation matrix. Thu, x- and y- components of incident light will experiment different refractive index and a phase difference are introduced with the length of LC layer. If the phase difference change to π/2, incident light’s polarization will rotate 90°, which means a polarization change for metasurface units.

**Figure 4:**
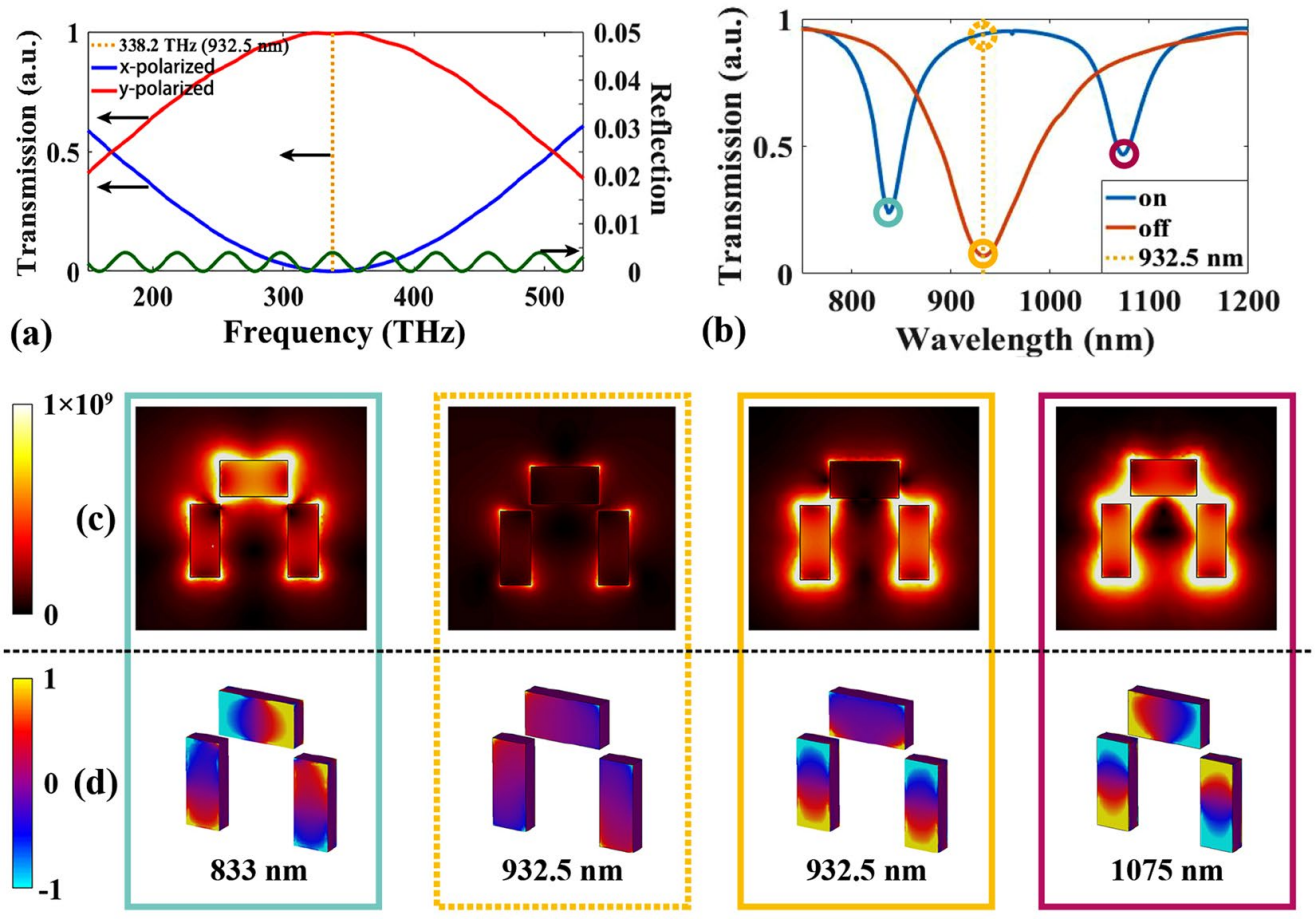
(**a**) Conversion for x-polarization incident light. (**b**) Spectrum comparison between the “on” and “off” states. (**c**) Electrical field distribution at the dip position of 932.5 nm in (**b**). (**d**) Corresponding charge distributions on the metasurface unit surface.

Figure 4(a) demonstrates that the output light polarization varies with the fixed thickness of the LC layer, from which we obtain the LC layer’s thickness of 2.3 μm, to achieve the largest modulation as a half wave plate (HWP) at 338.2 THz (932.5) nm, where the EIT metasurface resonates strongly. As demonstrated from the theoretical analysis, the incident light polarization can be rotated by 90° at 932.5 nm, which displays a total mode transition and an extremely low reflection at the same frequency point shown in Fig. 4(a).

### Electrically controlled switch and coupling analysis

The incident light oriented along the x-axis experiences a 90° polarization turn, i.e., along the *y*-axis after passing through the LC layer. The modulated light gives rise to the single resonance (spectral dip) at 932.5 nm, which is the “off” state in the absence of external voltage. If the external voltage is applied to the LC layer, no polarization modulation is introduced to the incident light and the light remains *x*-axis polarized when it reaches the metasurface and subsequently gives rise to another resonance. Two small spectral dips are observed on the two sides of the resonance at 932.5 nm where it is highly transparent and results in a significant transmission switch at this specific frequency.

Figure 4(b) exhibits the LC-metasurface transmission spectra in the “on” and “off” states. At 932.5 nm, the LC-metasurface transmission is 7.03% in the “off” state while reaching over 92.8% in the “on” state, which means that the modulation depth is ${m}_{d}=({T}_{on}-{T}_{off})/({T}_{on}+{T}_{off})=85.9 \%$ in the LC-metasurface. The transmission spectra of the EIT metasurface at different frequency ranges are mainly affected by the coupling efficiency, which is related to the field distributions and charge distributions shown in Fig. 4(c,d).

The strong coupling effect of the strips pairs gives rise to a much lower Rabi frequency than the one corresponding to the x-polarized incident light, which means that the doublet changes its profile to a single peak, as shown in Fig. 2(d,e) y-polarization line. The incident light gives rise to localized surface plasmons (LSPs) and couples into them at specific wavelength points (833 nm and 1075 nm for x-polarized incident light and 932.5 nm for the y-polarized incident light). Thus, the transmissions of the metasurface are much lower at these frequencies among others, which can be proven with the contrast of figures in yellow dashed and solid lines in Fig. 4(c,d). These two figures show the electrical field and charge distributions (normalized) respectively, at different spectral positions labeled by circles in Fig. 4(b). Figure 4(c,d) illustrate the electric field distributions and charge accumulations corresponding to the different wavelengths in Fig. 4(b). One can see clearly that at 932.5 nm the field distribution and charge distribution for the “on state” marked with the dashed yellow line is much weaker than that for the “off state” marked with the solid yellow line. This explains satisfactorily regarding the high transmission in the state marked with the dashed yellow line as well as the high absorption in the state marked with the solid yellow line. In the configuration with x-polarized incident light, the metasurface resonance is too weak to cause an absorption or a dip in spectra at the same frequency (932.5 nm), and the electric field distribution is extremely weak compared with that when the incident light is x-polarized, as shown in Fig. 4(c) (yellow dashed line). In addition, there are strong couplings of electric field and a remarkable charge distribution at the surface of the metasurface units between the radiative and dark elements at 833 nm and 1075 nm, which means that there are also two significant transmission dips at these two frequencies.

Furthermore, the transmission could be smaller than 7% at 932.5 nm in the “off” state if the number of layers in the metasurface structure increases; however, the high transparency in the “on” state must be sacrificed. In fact, achieving an even higher modulation depth may be challenging, owing to the deviations in fabrication of the system, for example, controlling the LC molecules. In our design, a simple, straightforward approach to conversion between the x- and y-polarized incident light is employed, in contrast to other methods that use a twisted LC structure, which is much more challenging to integrate with metasurfaces, and hence results in many more deviations in the polarization rotating process. It should be noted that the LC molecules in a thin layer close to the metasurface would deviate from the pre-alignment orientation because of the two orientations of metasurface unit strips being in the “off” state. Correspondingly, the LC molecules close to the brushed layer are subject to the pre-alignment more strongly than to the external electric orientation alignment. However, the degree of disordered molecules can be reduced by increasing the external electric field^33^. The impact of disordered molecules on light modulation is negligible not only because the strips in two axes can counterbalance the deviation forces but also because the dimensions of the metasurface units are much larger than the LC molecular scale. Meanwhile, with the LC tuning, the significant group velocity decay effect of EIT system developed our mind of dynamic slow-light device. In short, this design might be used in designing tunable metasurface systems and optical switches, wave front shaping, beam steering, dynamic slow-light, and in developing other active photonic devices.

## Discussion

We proposed a structure with a dynamic switching function, with two resonance modes of the EIT metasurface at near-IR wavelengths under x- and y-polarized incident light illuminations modulated by nematic LCs. This design results is an easy fabrication and takes advantage of LC processing technology in the display industries. The switchover between the “radiative” and the “dark” states and the decay rates of each state are closely related to the corresponding elements in the metasurface units. The absorption calculated with ${\chi }^{(1)}$, which is analyzed from the real and imaginary parts of the linear susceptibility with EIT atomic model, fits very well to our results. The electric field and units surface charge distributions are calculated, which shows a significant positive correlation with the system absorption. As a result, the LC-metasurface offers over 85% transmission difference and over 85.7% modulation depth in the NIR spectral region. In addition, the resonance response could be tuned with different modulation depth in different wavelength ranges.

## Methods

The frequency solver in Computer Simulation Technology (CST) Microwave Studio was employed to analyze the S parameters and the electric field of the designed structure. In our simulation, noble metal silver is chosen as a metasurface material^42^ owing to its excellent plasmonic properties in the NIR and visible regimes. A polarized electromagnetic wave was normally incident onto the system with its electric field along the *x*-direction, as shown in Fig. 1(a,b). In the simulations, periodic boundary conditions were selected to create the infinite metasurface in *x-y* plane and some open space was added along the z direction. The mesh density was set to be over 100 per metasurface unit length, which is much smaller than the size of the metasurface unit elements.

### Data availability

The data that support the findings of this study are available from the corresponding author upon reasonable request.

## Electronic supplementary material

Theory of EIT absorption spectrum
