# Supplementary material for: Liquid-Crystal-Based Electrically Tuned Electromagnetically Induced Transparency Metasurface Switch
Source: Sci Rep. 2017 Dec 12;7:17378. doi: 10.1038/s41598-017-17612-7 (PMC5727166; doi:10.1038/s41598-017-17612-7)
Supplement: Supplementary file 1 — Theory of EIT absorption spectrum [file 41598_2017_17612_MOESM1_ESM.doc]

**Liquid-Crystal-Based Electrically Tuned Electromagnetically Induced Transparency Metasurface Switch**

Hang Su,^1^† Hao Wang,^1^† Hua Zhao,^1^,* Tingyu Xue,^1^ and Jingwen Zhang,^1,2^,**

**Theory of EIT absorption spectrum**

The EIT-like metasurface can be described as a Λ-type three-level atom system and we calculated the absorption with different parameters to make an analogy with our simulation results. The energy levels are drawn in Fig.S1. In Fig. S1 (left), state$|1\rangle$is the ground state, state$|2\rangle$is a metastable state, which serves as the dark state in the system, and state$|3\rangle$is the excited state. Correspondingly, the radiative decay rate (Γ) of the dark state$|2\rangle$is much lower than that in $|3\rangle$($\Gamma_{3}\gg\Gamma_{2}$). For the EIT system, the pathway$|1\rangle\to|2\rangle$transition is always the dipole-forbidden thus electrons can be only excited from state$|1\rangle$to state$|3\rangle$and then trapped in the dark state, which is based on coherent population trapping^1^. The external laser field generates coherent coupling $|\text{a}^{\pm}\rangle$(Fig. S1 right) of the metastable state$|2\rangle$and provides another pathway through the doublet of dressed states ($|2\rangle\to|3\rangle$). Therefore, the electrons can be excited to state$|3\rangle$from the path$|1\rangle\to|3\rangle$with the coherence of the dressed states or from the path$|1\rangle\to|3\rangle\to|2\rangle\to|3\rangle$without that. On resonance, the atomic medium is opaque to the probe light, but with the presence of a strong control field, the system becomes transparent to the probe light.


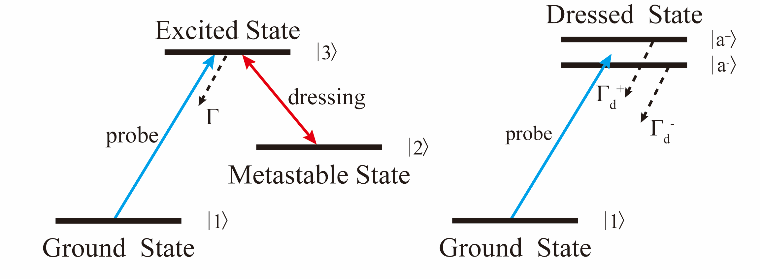


Fig. S1 Diagram of energy level transition for Λ-type EIT atom

In the interaction picture, the system’s time dependent interaction Hamiltonian describing the atom-laser coupling is^1^

(S1)

where $\Omega_{c}\left(t\right)$ is the Rabi frequency of the coupling field and $\Omega_{p}\left(t\right)$ is the probe field Rabi frequency.$\widehat{\sigma}_{ij}=|i\rangle\langle j|$is the atomic projection operator (*i*,*j*=1, 2, 3) and the $\Delta_{1}=\omega_{31}-\omega_{p}$ (the probe frequency) and $\Delta_{2}=\omega_{32}-\omega_{c}$ (the coupling frequency) denotes the detuning frequency from the excited state $|3\rangle$ to the other two states. The dynamics of laser-driven atomic systems are controlled by the master equation with the atomic density operator

(S2)

In Eq. (S2), the second and third terms describe the spontaneous emission from$|3\rangle$to$|1\rangle$or$|2\rangle$with rates $\Gamma_{31}$and$\Gamma_{32}$, respectively. In addition, energy-conserving dephasing processes with rates $\gamma_{3deph}$and$\gamma_{2deph}$ are introduced into the equation-presenting one of the fundamental time scales for practical EIT systems. We define $\Gamma_{3}=\Gamma_{31}+\Gamma_{32}$as the total spontaneous emission rate out of state$|3\rangle$and the coherence decay rates are$\gamma_{31}=\gamma_{3deph}+\Gamma_{3}$and$\gamma_{32}=\gamma_{3deph}+\Gamma_{3}+\gamma_{2deph}=\gamma_{31}+\gamma_{21}$.

We now focus on the linear susceptibility$\chi^{(1)}\left(-\omega_{p},\omega_{p}\right)$and the polarization $P\left(t\right)$with the off-diagonal density-matrix elements$\rho_{31}\left(t\right)$,$\rho_{32}\left(t\right)$and$\rho_{12}\left(t\right)$using the following equations and take$\rho_{11}\approx1$,

$\rho_{32}=\frac{i\Omega_{c}e^{i\Delta_{1}t}}{\left(\gamma_{32}+i2\Delta_{2}\right)}\rho_{12}$ (S3-1)

$\rho_{12}=-\frac{i\Omega_{c}e^{i\Delta_{2}t}}{\gamma_{21}+i2\left(\Delta_{2}-\Delta_{2}\right)}\rho_{13}$ (S3-2)

$\rho_{31}=\left[\frac{i\Omega_{c}e^{i\Delta_{1}t}}{\left(\gamma_{32}+i2\Delta_{2}\right)}+\frac{i\Omega_{c}e^{i\Delta_{2}t}}{\left(\gamma_{31}+i2\Delta_{1}\right)}\right]\rho_{21}$ (S3-3)

and the polarization generated in the atomic medium is

$\overrightarrow{P}\left(t\right)=-\sum_{i} \frac{\left\langle e\overrightarrow{r}_{1}\right\rangle}{V}=\frac{N_{atom}}{V}\left[\overrightarrow{\mu}_{13}\rho_{31}e^{-i\omega_{31}t}+\overrightarrow{\mu}_{23}\rho_{32}e^{-i\omega_{32}t}+c.c.\right]$ (S4)

Without losing generality, we assume the photons from the Λ-type atoms are all along z-axis of the designed metasurface structure and obtain $\overrightarrow{\mu}_{13}=\mu_{13}\widehat{z}$, $\overrightarrow{\mu}_{23}=\mu_{23}\widehat{z}$ and $P_{z}\left(t\right)=P\left(t\right)$,


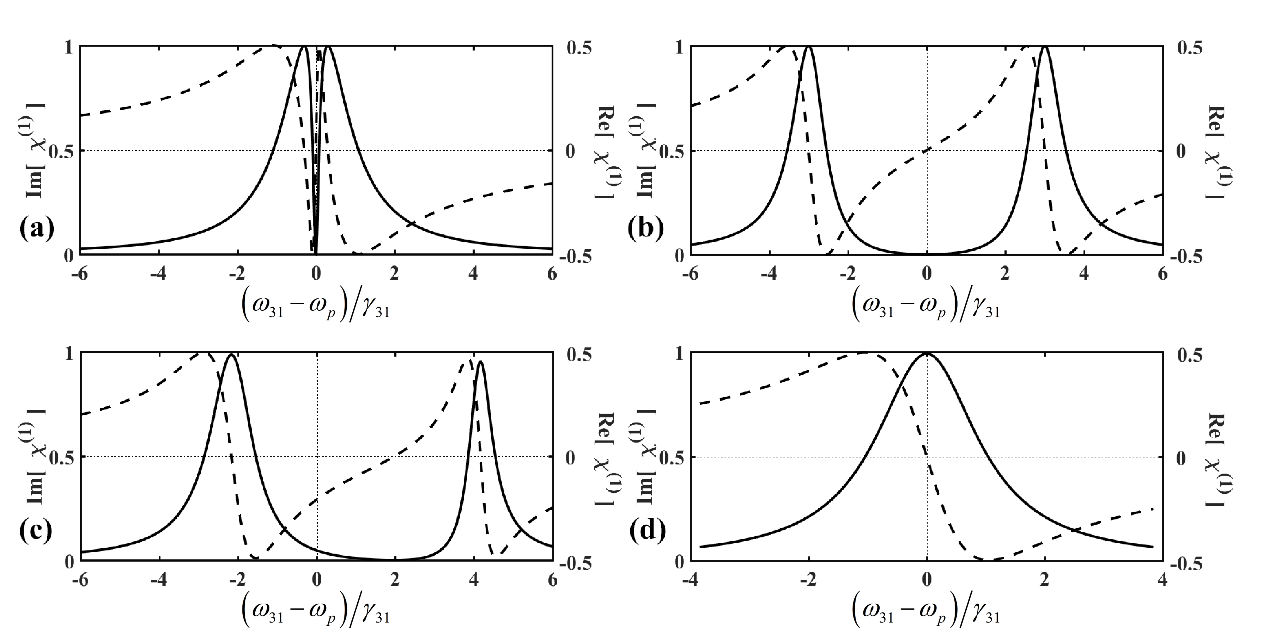


Fig.S2 (a)-(d) Imaginary part (solid line) and real part (dash line) of with different parameters selected: (a),and; (b),and; (c),and; (d),and;

thus the linear susceptibility can be obtained by

(S5)

where$\xi=\frac{N_{atom}}{V}$describes the atomic density, the single photon detuning as$\Delta=\Delta_{1}=\omega_{31}-\omega_{p}$and the two-photon detuning as$\delta=\Delta_{1}-\Delta_{2}$. The linear susceptibility$\chi^{(1)}\left(-\omega_{p},\omega_{p}\right)$in Eq. (S5) is divided into real and imaginary parts, which are in direct proportion to the system linear refractive index$n_{0}=\left(1+\mathrm{Re}\left[ \chi^{(1)}\left(\omega\right)\right]\right)^{\frac{1}{2}}$and linear absorption coefficient $\alpha_{0}=\frac{k_{0}}{n_{0}}\mathrm{Im}\left[ \chi^{(1)}\left(\omega\right)\right]=\frac{\omega}{cn_{0}}\mathrm{Im}\left[ \chi^{(1)}\left(\omega\right)\right]$, respectively. The transmission of metasurface-LC layer is related to the absorption and reflection of the system, which are controlled by$\alpha_{0}$and$n_{0}$, namely,$\chi^{(1)}\left(\omega\right)$. From Eq. (5), the imaginary part of$\chi^{(1)}\left(\omega\right)$is controlled by$\Omega_{c}$,$\Delta$,$\gamma_{31}$and$\gamma_{21}$. In Fig S2, for an ideal condition, $\Omega_{c}\ll\gamma_{31}$and electrons are trapped in state$|2\rangle$and thus$\gamma_{21}=0$, which results in a typical line type in Fig. S2(a). If $\Omega_{c}>\gamma_{31}$, the doublet will separate away from each other as shown in Fig. S2(b). However, the detuning$\Delta_{2}$cannot be neglected in our design, thus two peaks are asymmetrical, which is shown in Fig. S2(c).

When the incident light mode is changed from x-polarization to y-polarization, the “dark state” element changes into the ordinary “radiative state” in the metasurface and vice versa. Thus, the electrons cannot be trapped in the dark state$|2\rangle$for a long time due to the small size of “dark state”. In this situation, the decay rate$\gamma_{21}$can no longer be treated as 0 in Eq. (S5) and this results in a quite different line type shown in Fig. S2 (d). Under the condition of incident polarization is along the *x*-axis, the radiative plasmonic strip offers a radiation state, which strongly couples with the incident field$E_{0}=\widetilde{E}_{0}\left(\omega\right)e^{-i\omega t}$and the dark elements offer a dark plasmonic state, which weakly couples to the incident light. Correspondingly, under the condition of *y*-axis polarization, the radiative element and dark elements exchange their functions and generate a completely different spectrum response with the x-polarization incident light. With the specially chosen spatial position and dimensions, the metasurface creates a strong spectral dip at around 500 THz under illumination of the x-polarization incident light and two dips under the y-polarization. It should be noted that the scales in Fig. S2 is normalized.

1 Fleischhauer, M., Imamoglu, A. & Marangos, J. P. Electromagnetically induced transparency: Optics in coherent media*. Rev. Mod. Phy*s**.** 77, 633 (2005).
